# Quantitative *In Vivo* Detection of *Chlamydia muridarum* Associated Inflammation in a Mouse Model Using Optical Imaging

**DOI:** 10.1155/2015/264897

**Published:** 2015-11-18

**Authors:** Manishkumar Patel, Shu-An Lin, Melissa A. Boddicker, Christopher DeMaula, Brett Connolly, Bohumil Bednar, Jon H. Heinrichs, Jeffrey G. Smith

**Affiliations:** ^1^Department of Imaging, Merck Research Laboratories, Merck and Co., 770 Sumneytown Pike, West Point, PA 19438, USA; ^2^Department of Vaccine Research, Merck Research Laboratories, Merck and Co., 770 Sumneytown Pike, West Point, PA 19438, USA; ^3^Department of Pathology, Merck Research Laboratories, Merck and Co., 770 Sumneytown Pike, West Point, PA 19438, USA

## Abstract

*Chlamydia trachomatis* is a bacterial sexually transmitted disease with over 1.3 million cases reported to the CDC in 2010. While Chlamydia infection is easily treated with antibiotics, up to 70% of infections are asymptomatic and go untreated. The current mouse model relies on invasive upper genital tract gross pathology readouts at ~60–80 days postinfection. High throughput optical imaging through the use of biomarkers has been successfully used to quickly evaluate several disease processes. Here we evaluate Neutrophil Elastase 680 (Elastase680) for its ability to measure *Chlamydia muridarum* associated inflammation in live mice using fluorescence molecular tomography (FMT) and *In Vivo* Imaging System (IVIS). Optical imaging was able to distinguish with statistical significance between vaccinated and nonvaccinated mice as well as mock-challenged and challenged mice 2 weeks after challenge which was 9 weeks sooner than typical gross pathological assessment. Immunohistochemistry confirmed the presence of neutrophils and correlated well with both *in vivo* and *ex vivo* imaging. In this report we demonstrate that Elastase680 can be used as a molecular imaging biomarker for inflammation associated with chlamydial infection in a mouse model and that these biomarkers can significantly decrease the time for pathology evaluation and thus increase the rate of therapeutics discovery.

## 1. Introduction

*Chlamydia trachomatis* is the most common notifiable sexually transmitted infection with approximately 1.4 million cases reported to the Centers for Disease Control and Prevention in 2011 [1]. While Chlamydia urogenital infection can be effectively treated with antibiotic therapy, a majority of infections remain undetected and therefore go untreated. Chlamydia urogenital infection can lead to serious chronic disease consequences including urethritis/cervicitis at the mucosal site of infection, ascending infection resulting in pelvic inflammatory disease (PID), ectopic pregnancy, and tubal factor infertility (TFI) [2, 3].

Development of a vaccine for Chlamydia is widely considered the most effective approach for the prevention of infection and the associated disease sequelae. Well-characterized tools to support the effective evaluation of therapeutic intervention are prerequisite to the development of a Chlamydia vaccine and this includes tools for the evaluation of immune responses induced by vaccination and their impact on the course of infection. Equally important is an effective evaluation of the impact these experimental interventions have on the development of genital tract pathology.

Mouse models of reproductive tract infection with* Chlamydia muridarum* have proven useful for the study of immune mediators associated with bacterial clearance, development of disease pathogenesis, and the evaluation of vaccination efficacy [4–10]. Infection with* C. muridarum* in mice results in short-lived immunity that has been demonstrated to be dependent upon T-cell associated immune mechanisms for clearance, it leads to injury of the oviducts and reduction in fertility similar to the human condition, and it has been shown that repeated infection can elicit even more severe disease sequelae such as pelvic inflammatory disease (PID) and tubal factor infertility (TFI) like disease [10–13].

The exact mechanisms leading to tissue damage are not well understood. A cellular pathogenesis paradigm by which ineffective bacterial clearance (Chlamydia persistence) leads to release of proinflammatory cytokines from infected cells and resultant tissue damage is one prominent theory [14]. Alternatively, there is an immunological pathogenesis paradigm where repeated infection increases severity of the inflammatory response resulting in tissue damage at the focal sites of infection [14, 15]. It may be that both of these paradigms play some role in Chlamydial reproductive tract pathogenesis.

One hallmark of acute chlamydial genital tract infection in the mouse model is a large neutrophilic infiltrate, with both recruitment and longevity of this influx linked to severity of oviduct pathology [16, 17]. This parallels the observation in humans of elevated cervical sample neutrophil defensin levels in patients with Chlamydia-associated endometritis compared with uninfected subjects [18]. Further, primary infection with either wild type or plasmid-deficient* C. muridarum* strains followed by secondary challenge with a wild type strain resulted in a reduced frequency of neutrophil accumulation in the cervix and oviducts compared with primary wild type strain challenge [19]. Sterilizing immunity was not achieved by the prior challenge, yet there was a measured reduction in the inflammatory response and resultant tissue pathology. Taken together, these findings suggest that measurements of neutrophil infiltration following challenge would correlate with Chlamydia-related pathology in the mouse model, and measurement at key time points could be used to support the evaluation of vaccines or other novel therapeutic interventions.

Neutrophil elastase is a serine protease produced by activated neutrophils during the inflammatory response and has been used as an effective biomarker target [20, 21]. In this study, we evaluated the near-infrared optical imaging probe, Neutrophil Elastase 680 FAST (Elastase680) for the longitudinal evaluation of acute inflammation following primary Chlamydia infection and with Chlamydia challenge following vaccination. Near-infrared molecular imaging biomarkers have been used in the detection of inflammation in various animal models [22]. Use of these agents in conjunction with fluorescence molecular tomography (FMT) and computer tomography (CT) was applied for* in vivo* measurement and quantitative evaluation of the levels of inflammation in mouse models of atherosclerosis [23, 24]. In this study, signal from the Elastase680 probe was evaluated using both* ex vivo* (imaging of isolated genital tract tissues) and noninvasive live animal imaging approaches in comparison with standard histological tissue evaluation and immunohistochemical (IHC) tissue staining.

## 2. Methods

### 2.1. Ethics Statement

All experiments were approved by the Merck Institutional Animal Care and Use Committee (IACUC). All animal studies were conducted according to The Guide for the Care and Use of Laboratory Animals [25] and veterinary care was given to any animals requiring medical attention.

### 2.2. Animal Immunizations and Challenge

Albino C57B6 mice were purchased from Charles River and placed on alfalfa free diet (Dyets Inc., PA). Chlamydiae were propagated as described previously [26]. Briefly, mice were immunized with live* Chlamydia muridarum* Elementary Bodies (EB) at 1 × 10^6^ EB/mouse in sucrose-phosphate-glutamate (SPG) buffer by the intraperitoneal (ip) route. Immunizations were administered on days 0, 20, and 30. At approximately 2 weeks following the last immunization, progesterone (medroxyprogesterone acetate, Depo-Provera; Pfizer; New York, NY) was administered subcutaneously (2.5 mg/dose) at 10 and 3 days before challenge. Mice were challenged intravaginally (approximately 1 month following last immunization) by direct instillation of 10 *μ*L of SPG containing 1 × 10^4^ *C. muridarum* EBs. The vaginal vault and ectocervix were swabbed using a microfiber swab (Fisher, Pittsburgh, PA) on days 7, 11, 14, 18, and 21 after challenge or some combination of these time points.

Swabs were placed into a 1.5 mL tube containing two sterile glass beads (5 mm diameter) and 300 *μ*L of chlamydia isolation medium (Trinity Biotech, Berkeley Heights, NJ) on ice. Bacteria were eluted from the swabs and separated from cells by vortexing for 60 s. Eluted bacteria (100 uL) were plated onto a processing cartridge containing 100 uL of PBS and stored at −70°C until DNA extraction using the MagNA Pure 96 DNA and Viral NA small volume kit on the Roche MagNA pure machine (Roche, Indianapolis, IN). Real-time quantitative polymerase chain reaction (RT-qPCR) was performed to confirm the presence of infection as described previously [26].

### 2.3. Optical Imaging

At indicated time points, mice were shaved and Nair (Church and Dwight, Princeton, NJ) was used to remove residual hair on the ventral and dorsal abdominal region. Mice were injected intravenously with 2 nmol of Neutrophil Elastase 680 FAST (Elastase680, Perkin Elmer, Waltham, MA). Six hrs after injection mice were anesthetized with 2-3% Isoflurane (Isothesia, Henry Schein Animal Health, Dublin, OH) in O_2_ at a rate of 1 L/min, placed in the FMT holder, and then imaged in the dorsal position using an FMT 2500 imaging system (Perkin Elmer). In some studies, after live animal* in vivo* imaging, the mice were euthanized according to the AVMA Guidelines for the Euthanasia of Animals: 2013 Edition [27] and reproductive tracts harvested and imaged with the IVIS Spectrum (Perkin Elmer) system using 680 nm excitation and 750 nm emission filter sets before being processed for histomorphologic and immunohistochemical evaluation. Analysis was performed using Truequant (FMT) and Living Image (IVIS) software (Perkin Elmer, Waltham, MA). FMT data are presented as the amount of probe in pmol present in regions of interest (ROI). IVIS data are presented as Radiant Efficiency (photons/s)/(*μ*W/cm^2^) which is a measure of photon flux from a source, normalized by exposure time, area of emission, and solid angle of the detector. Statistical analysis was performed using either one-way or two-way ANOVA on SigmaPlot software (Systat, San Jose, CA).

### 2.4. Micro-CT Imaging

Mice were imaged with micro-CT after FMT imaging. Mice were maintained under anesthesia and moved to the micro-CT scanner while in the FMT holder to maintain position. Micro-CT images were acquired using the eXplore Locus Ultra (TriFoil Imaging, Chatsworth, CA) with X-ray settings of 80 kV and 50 mA using the 16 sec anatomical protocol, which collects 1000 projections in a single 360° gantry rotation. The projection data for each scan was reconstructed using the AxRecon reconstruction system (Scanco, Switzerland). The reconstructed image slices consisted of 100 *μ*m isotropic voxels scaled to Hounsfield units (HU). The FMT holder contains fiducial markers visible in both FMT and micro-CT datasets. FMT data were then exported to DICOM format using Truequant. Both micro-CT and FMT datasets were imported into Amira software (Visage Imaging, Andover, MA) which was used to coregister the data. A pseudo color scale based on fluorophore concentration was applied to the FMT data for better visualization.

### 2.5. Histopathology and Immunohistochemistry

After the final imaging session, tissues were fixed in 10% neutral buffered formalin for 24 hr, then processed, and embedded in paraffin by routine methods for histomorphologic evaluation and immunohistochemistry analysis. Replicate four-micron thick sections were cut and mounted onto positively charged slides. One slide set was stained with hematoxylin and eosin (H&E) according to routine methods for histopathologic examination. A second set was further processed for IHC staining. Briefly, heat-induced epitope retrieval (HIER) was performed with a commercial buffer (DIVA Decloaker; Biocare Medical, Concord, CA) using a pressure cooker. After cooling the slides were washed in dH_2_O, rinsed in phosphate buffered saline containing 0.1% Tween 20 (PBST, pH 7.2), and loaded onto an automated IHC stainer (intelliPATH, Biocare, Concord, CA). Endogenous peroxidase was blocked with 3% H_2_O_2_ for 20 min, slides were washed in PBST (3 × 3 min), and then incubated for 30 min in serum free blocking buffer (Background Sniper; Biocare). Slides were incubated in 1.0 ug/mL rat-anti mouse Ly-6B.2 alloantigen to detect neutrophils (MCA771GA; AbD Serotec, Raleigh, NC) for 1 hr and washed in PBST as before. The bound antibody was detected using a rat-on-mouse HRP-polymer kit (RT517; Biocare) according to the manufacturer's instructions. After additional PBST washes the slides were incubated with diaminobenzidine substrate (DAB) for 5 min followed by counterstaining with hematoxylin.

Histomorphologic evaluation was conducted by a trained pathologist and results were peer-reviewed. Histomorphologic diagnoses were made separately for ovary and surrounding tissues, oviduct, uterus, cervix, and vagina and graded semiquantitatively on a scale of + to +++++ according to routine procedures (+ = very slight, ++ = slight, +++ = moderate, ++++ = marked, and +++++ = severe).

## 3. Results

### 3.1. Elastase Signal Localization and Time Course Following Chlamydia Genital Tract Infection

**Figure 1:**
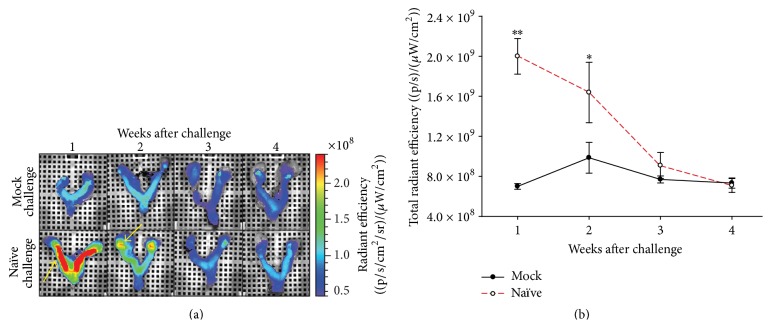
Elastase signal localization and time course following Chlamydia genital tract infection. (a) Representative pseudoscaled images of mouse reproductive tracts after Elastase680 injection. All images are scaled to the same color bar with red indicating highest signal intensity and purple being the lowest. The yellow arrows point to areas of high Elastase680 signal, the uterine horns in week 1, and the ovary area in week 2. (b) Quantitation of images in part (a). Manual regions of interest (ROI) were drawn over the entire reproductive tract of *N* = 5 mice/group and quantified as Total Radiant Efficiency (photons/s)/(*μ*W/cm^2^), a sum of fluorescent pixels within the ROI. Closed symbols represent mock-challenged mice and open symbols represent naïve/challenged animals. The term naïve is used here to be consistent with terminology used later in the paper to distinguish between vaccinated and nonvaccinated (naïve) animals. ^*∗*^ *p* = 0.008; ^*∗∗*^ *p* < 0.001.

Mice were either challenged intravaginally with* C. muridarum* (naïve/challenged) as described or received a mock challenge with PBS. The term naïve is used here to be consistent with terminology used later in the paper to distinguish between vaccinated and nonvaccinated (naïve) animals. Seven, 14, 21, and 28 days following challenge, separate groups of mice were injected with Elastase680 and then euthanized after the washout period. The genital tracts were harvested and imaged* ex vivo*. One week following infection the Elastase probe signal was detected primarily in the uterine horns in Chlamydia-infected mice and not in PBS mock-infected mice. At 14 days the probe signal was detected primarily in the oviduct area, and by 21 days after challenge probe signal detection was reduced in all genital tract regions (Figure 1(a)). Region of interest (ROI) analysis was performed over the entire genital tract and used to quantitate the neutrophil response over the region. Seven and 14 days after challenge the quantified signal was significantly higher in Chlamydia-infected mice than in mock-infected mice (Figure 1(b)).

### 3.2. *In Vivo* Live Animal Imaging with Elastase680 Is a Biomarker at the Oviduct Level

**Figure 2:**
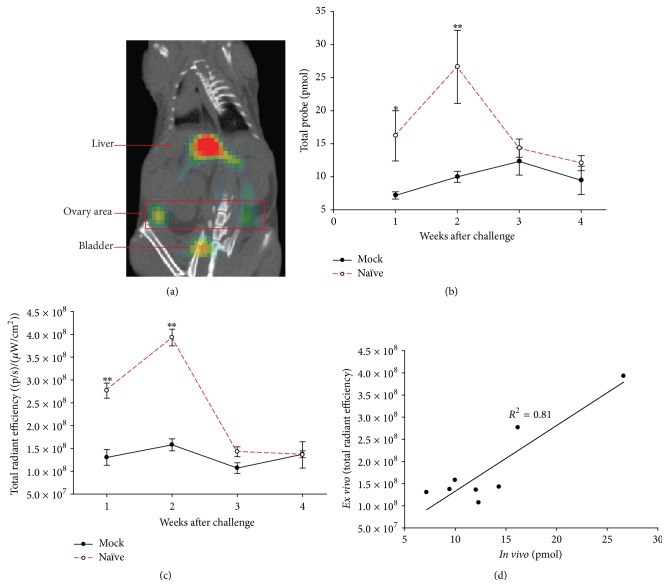
*In vivo* live animal imaging with Elastase680 correlates at the oviduct level. (a) FMT data overlaid onto a uCT scan of the same mouse. The red box indicates the ROI drawn for analysis. This area correlates with the location of the ovary/oviduct which sits below the kidneys as seen in the uCT. Other areas of signal are labeled. These include the liver and bladder which are clearance organs for Elastase680. (b) Quantitation of FMT signal. Box ROIs were drawn over the ovary area of *N* = 5 mice/group as indicated in (a) and quantified as total probe or amount of fluorescence in pmol. Closed symbols represent mock-challenged mice and open symbols represent naïve/challenged animals. (c) Quantitation of only the ovary region from Figure 1. Circular ROIs were drawn over the ovary region. The signal from each ovary was then combined for each sample and quantified as Total Radiant Efficiency. ^*∗*^ *p* = 0.026; ^*∗∗*^ *p* < 0.001. (d) Correlation of* in vivo* and* ex vivo* signal.

Challenged and mock-challenged mice were also scanned using noninvasive imaging (live* in vivo* imaging).  Figure 2(a) shows an example of an animal scan coregistered with a microCT scan of the same animal; the image shows the raw signal without any processing. Fiducial markers in the animal holder allowed for coregistration of the two modalities. Signal from the uterine horns during* in vivo* imaging was masked by excreted probe in the bladder and therefore analysis could only occur at the ovary/oviduct level. Signal intensity in this area peaked at approximately 14 days after challenge and was significantly higher than signal in a similar area in mock-infected animals (Figure 2(b)). ROI analysis on* ex vivo* images of the ovary/oviduct also showed signal intensity peaking 14 days after challenge (Figure 2(c)). There was a good correlation between* ex vivo* imaging and* in vivo* imaging (Figure 2(d)).

### 3.3. Elastase680 Is Able to Distinguish between Nonvaccinated and Vaccinated Animals

**Figure 3:**
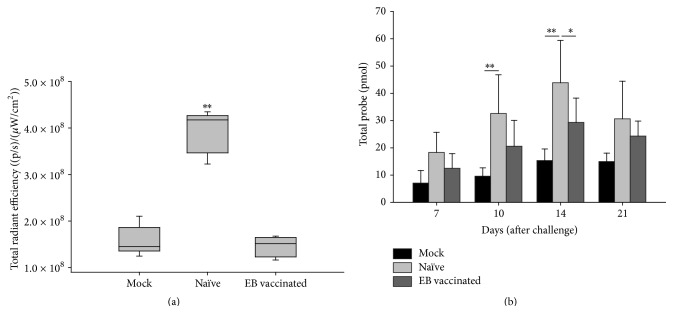
Elastase680 is able to distinguish between nonvaccinated and vaccinated animals. (a)* Ex vivo* ROI analysis on the ovary area of reproductive tracts (*N* = 5/group) 14 days after challenge. At 14 days there was a statistically significant difference between vaccinated animals and naïve (nonvaccinated) animals. (b) At the indicated time points mice (*N* = 10/group) were imaged* in vivo* and analysis on the ovary region was performed as described in Figure 2. At day 10 there was a statistically significant difference between mock-challenged and naïve/challenged groups only while on day 14 there was a significant difference in both mock-challenged and vaccinated groups versus naïve/challenged group. ^*∗*^ *p* = 0.03; ^*∗∗*^ *p* < 0.001.

Next we performed longitudinal imaging on nonvaccinated and vaccinated mice to determine if noninvasive imaging can differentiate between these groups. Mice were either mock challenged with PBS (mock), challenged (naïve) with* C. muridarum,* or vaccinated with EBs (EB vaccinated) and then challenged with* C. muridarum *intravaginally as described above. Five mice from each group were euthanized at two weeks after challenge for* ex vivo* analysis while the remainder (*N* = 10) were allowed to progress to genital tract gross pathology development and observational scoring (11 weeks postinfection). Day 14 after challenge was chosen for* ex vivo* evaluation because previous studies showed this to be the peak time point for the Elastase680 signal. At 14 days after challenge there was a statistical difference in quantified signal between vaccinated and naïve mice using* ex vivo* analysis on the ovary/oviduct region of the reproductive tracts with the Elastase680 probe (Figure 3(a)). However, this differentiation did not occur until day 14. In the longitudinal group, at earlier time points there was an observable trend but there was no statistical difference in the Elastase680 signal between naïve and vaccinated mice (Figure 3(b)). Both sets of mice had a higher Elastase680 signal than mock-challenged mice although vaccinated mice had a lower signal intensity than naïve mice at both day 7 and day 10 after challenge. Eighty days after challenge 80% of the naïve mice developed gross pathology while only 25% of the vaccinated mice developed gross pathology (Table 1). As there is a close association between neutrophil infiltration and the level of late stage pathology [17, 28] the findings at day 80 could have been predicted at day 14 when the differentiation between the treatment groups using Elastase680 signal was observed.

### 3.4. Immunohistochemistry and Histopathology Confirm Elastase680 Signal

To confirm that neutrophils were present in each section of the genital tract identified by imaging, we performed histopathology to estimate semiquantitatively the levels of acute inflammation on excised genital tracts.  Table 2 summarizes the notable histopathological findings related to* C. muridarum* challenge in mice. A detailed table of pathological findings can be found in the supplemental sections (Table S1 in Supplementary Material available online at http://dx.doi.org/10.1155/2015/264897). Prior to 14 days, the majority of infected mice had histomorphologic changes that were acutely inflammatory in nature and localized primarily in the oviducts and/or the connective tissues around the oviducts and ovaries. Acute inflammation in the uterus was very slight to moderate and present mostly in the endometrium of the uterine horns. The acute inflammatory infiltrate was comprised primarily of neutrophils and lower levels of lymphocytes and macrophages in all areas. Approximately 14 days after challenge, similar acute inflammatory changes were present with a high incidence in the oviducts and/or periovarian areas in infected mice. At 21 days after challenge, inflammation in the periovarian tissues, oviducts, and uterus was more chronic in nature, with mostly lymphocytes and substantially fewer neutrophils infiltrating the tissues. Mock-infected mice had no notable histomorphologic changes in the reproductive tract as expected.

**Figure 4:**
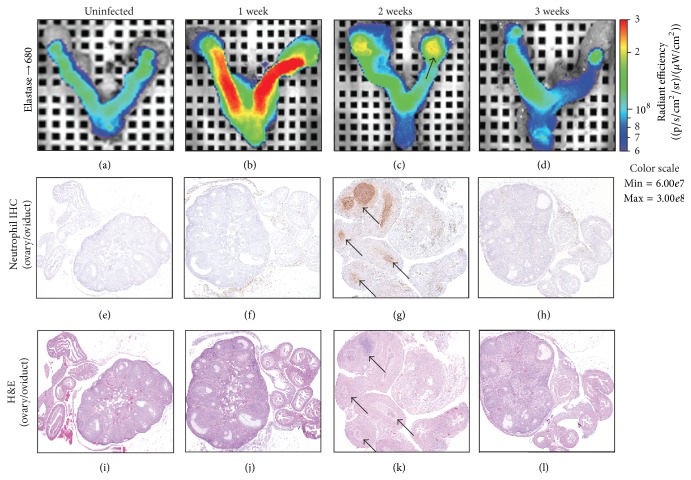
Elastase680 probe signal, Ly 6B.2 neutrophil immunohistochemistry, and H&E staining in the genital tracts of albino C57B6 mice following intravaginal challenge with* C. muridarum*. Elastase680 probe signal, Ly 6B.2 neutrophil immunohistochemistry, and H&E staining in the genital tracts of albino C57B6 mice following intravaginal challenge with* C. muridarum*. Mock-challenged animals show minimal Elastase680 signal (a) and no evidence of inflammation (e, i). At 1 week after challenge, Elastase680 signal is present in the uterine horns (b) and there is no acute inflammation present in the ovaries or oviducts (f, j). At 2 weeks after challenge the Elastase680 signal is primarily present within the ovary/oviduct region, corresponding to areas of an acute inflammatory cell infiltrate (arrows) comprised primarily of neutrophils (g, k). By 3 weeks after challenge the Elastase680 signal was reduced (d) to levels seen in mock-challenged mice and the neutrophilic infiltrate had subsided (h, l).

To further characterize the neutrophilic component of the inflammatory response observed by routine histomorphologic evaluation, we performed immunohistochemistry using an antibody (Ly6B.2) that specifically labels murine neutrophils (Figure 4, Table 3). Approximately seven days after challenge aggregates of Ly 6B.2 immunoreactive neutrophils were most predominant in the uterine horns and oviducts and in some of the surrounding stroma in the infected mice. As the infection progressed through 14 days the most abundant neutrophil aggregates were found to be associated with the oviducts and adjacent tissues as indicated by the severity score in Table 3. At day 21 after challenge, the number of Ly 6B.2 positive neutrophils was greatly reduced compared to earlier time points, consistent with the histopathology evaluation of routinely prepared sections. Both the percentage and intensity (percent area of positive staining) of Ly6B.2 were reduced in vaccinated mice verses naïve mice at all time points. Consistent with the* in vivo* imaging findings, the vaccinated mice did have neutrophil infiltration in the oviduct area at day 7 and day 10 after challenge and the level of neutrophil infiltration dropped off significantly at day 14. Overall immunohistochemistry and histopathology results correlated well with Elastase680 imaging results. Thus, noninvasive imaging using the neutrophil biomarker Elastase680 accurately reflected neutrophil infiltration in the ovary/oviduct area.

## 4. Discussion

Previous studies have shown gross pathology or histological evaluation following Chlamydia reproductive tract challenge and the effectiveness of live Chlamydia EB vaccination in preventing the damage associated with this infection [4–8, 11, 13, 19]. However, evaluation of Chlamydia-induced gross pathology and the effect of interventional strategies in the mouse model have been difficult to quantify, making effective comparison and development of treatments slow and challenging. Past approaches have included applying gross pathological observational scoring of hydrosalpinx and/or uterine dilatation at time points distal from challenge (70–80 days) or microscopic histopathology evaluation at earlier time points. Both of these approaches are terminal procedures capturing a point in time measurement and are subjective assessments. Development of a less subjective and quantitative method for tracking responses to* C. muridarum* early in the course of infection without the need to euthanize animals would allow faster and more thorough evaluation of* C. muridarum* pathogenesis and response to interventional therapies than is currently available. Furthermore, the longitudinal nature of these studies would allow for better interpretation of data, as each animal can act as its own control. In this study we have shown that Neutrophil Elastase 680 (Elastase680) based fluorescence molecular tomography (FMT) can be used to track inflammatory responses to* C. muridarum* in the mouse model early in the course of infection. We further demonstrated utility in application to Chlamydia vaccine evaluation.

Elastase680 is a noninvasive imaging neutrophil biomarker and has been previously shown to track the presence of infiltrating neutrophils associated with acute inflammation [20, 21]. Other studies have labeled Chlamydial strains with either a fluorescent dye [29] or bioluminescent protein [30] to visualize Chlamydial infection in mice. These studies provided the ability to track the bacteria in live animals but do not predict downstream pathology. This is an important parameter for vaccine discovery especially since different mouse strains have different responses to* C. muridarum* infection [31]. Studies have shown a close association of downstream pathology and the level of neutrophil recruitment in mouse models of* C. muridarum* infection [17, 28].* In vivo* imaging using Elastase680 allowed for noninvasive monitoring of neutrophil infiltration associated with* C. muridarum* challenge. Peak signal in the upper genital tract of naïve/challenged animals occurred two weeks after challenge before declining to mock challenge levels by four weeks after challenge. This acute inflammatory time course correlated with neutrophil infiltration as determined by histopathology and immunohistochemistry and aligned with previous reports showing that peak inflammatory response occurs about two weeks following* C. muridarum* challenge [16, 17, 19].

One of the limitations of using Elastase680* in vivo* in this model is its clearance through the bladder where the probe accumulates before being excreted from the body. This excess signal obscures any detectable signal in the uterine horns. However, signal located in the uterine horns was easily detectable upon removal of the genital tract and analysis* ex vivo*. At one week after challenge Elastase680 signal is first detectable in the uterine horns of naïve/challenged animals and neutrophil presence was confirmed with histopathology and immunohistochemistry and supported by previous studies [16]. However,* in vivo* evaluation of the oviduct region was ultimately sufficient for the purposes of tracking* C. muridarum* infection and predicting outcome in the mouse model.

Elastase680 was also able to distinguish between EB vaccinated and unvaccinated mice. At two weeks after challenge in EB vaccinated mice, little or no Elastase680 signal was present in the oviduct region. Again, this was confirmed with histopathology and immunohistochemistry which demonstrated few or no neutrophils present. However, at earlier time points EB vaccinated animals had showed a transient increase in Elastase680 signal, indicating that there was some transient neutrophil infiltration in EB vaccinated animals. In animals that were longitudinally followed with imaging and allowed to progress to pathology at day 80 after challenge there was no pathology observed in the EB vaccinated animals. Studies have linked the duration of time neutrophils are present with subsequent pathology in mouse models [17, 28]. Noninvasive imaging may enhance the study of this link by monitoring the same animal over time before pathological evaluation. In addition, mice can be kept alive for fertility studies and Elastase680 could serve as an earlier biomarker for vaccine efficacy. An additional benefit of the use of longitudinal, noninvasive imaging is a reduction in the number of mice required for studies, since mice do not need to be euthanized at time points during the course of the study.

Optical imaging using Elastase680 to monitor acute inflammation caused by* C. muridarum* infection can be applied in a consistent manner to reduce study time and animal numbers or to evaluate individual mice over time. Recently it was reported that plasmid free* C. muridarum* can also cause hydrosalpinx in some strains of mice [32]. Since hydrosalpinx is a chronic pathology that results from acute inflammation it is possible that Elastase680 could be used to aide in discovery of the levels of hydrosalpinx caused by different strains of* C. muridarum*. More importantly imaging is quantitative and not subjective. Not only can imaging be applied to investigate reduction in pathology with vaccination, but also it may have utility in monitoring the possibility of enhanced pathology with ineffective vaccination as was observed with some early studies in both nonhuman primates and human volunteers with chlamydia EB vaccines [33–35]. Imaging with Elastase680 and other developing immunological probes provides new biomarker tools to support the effective evaluation of pathogenesis following chlamydial infection and the effect of vaccination.

## Supplementary Material

A detailed histopathologic summary of findings in reproductive tracts of female mice infected with *C. muridarum*. Mice were sacrificed at the indicated time point and the reproductive tracts were prepared for histopathology assessment as described in the methods. The data presented here is a compilation of several studies. The numbers indicate the percentage of mice that had findings. Severity was graded semi-quantitatively on a scale of 1 to 5 according to routine procedures (1=very slight, 2=slight, 3=moderate, 4=marked, and 5=severe). Acute inflammation was mostly neutrophilic while chronic inflammation was lymphocytic in nature.

## Figures and Tables

**Table 1:** Gross pathology at day 80.

Group	% of mice with pathology
Mock-challenged	0%
Naïve/challenged	80%
EB vaccinated/challenged	25%

Mice were euthanized and the upper genital tracts were assessed for Luminal distention of the uterine horns and dilatation of the oviducts. Presence of either the Luminal distention of the uterine horns or dilatation of the oviducts resulted in a positive pathology score for the animal. The % pathology score was based on an *n* of 20 animals per group.

**Table 2:** Histopathology summary.

Incidence (%) and severity of histomorphologic findings in mice infected with *C*. *muridarum*								

Days postinfection	7		10		14		21	

Vaccination status	N	V	N	V	N	V	N	V

# Mice evaluated	15	5	5	5	25	9	15	5

Oviduct (UGT)								
Inflammation-acute	60	40	80	60	96	—	7	—
Severity	++	+++	++++	+++	++++		+	
Inflammation-chronic	—	—	—	—	—	11	87	40
Severity						+	++	+
Uterine horns (LGT)								
Inflammation-acute	87	60	100	80	92	—	—	—
Severity	+	+	++	+	++			
Inflammation-chronic	—	—	—	—	—	—	87	40
Severity							+++	++

Mice were euthanized at the indicated time point and the reproductive tracts were prepared for histopathology assessment as described in Section 2. The data presented here is a compilation of several studies. The numbers indicate the percentage of mice that had findings. Severity was graded semiquantitatively on a scale of + to +++++ according to routine procedures (+ = very slight, ++ = slight, +++ = moderate, ++++ = marked, and +++++ = severe). Acute inflammation was mostly neutrophilic while chronic inflammation was lymphocytic in nature.

N: not vaccinated; V: EB vaccinated; —: no noteworthy change; UGT: upper genital tract, and LGT: lower genital tract.

**Table 3:** Immunohistochemistry summary.

Incidence (%) and severity of immunohistochemical (Ly6B.2) findings in mice infected with *C*. *muridarum*								

Days after challenge	7		10		14		21	

Vaccination status	N	V	N	V	N	V	N	V

# Mice evaluated	15	5	5	5	25	9	15	5

Oviduct								
Ly6B.2 positive	80	40	80	60	96	0	87	20
Severity	++	+++	++++	+++	++++		+	+
Uterine horns								
Ly6B.2 positive	100	60	100	80	88	0	47	20
Severity	++	++	++	+	++		+	+

The same tissues as in Table 2 were also processed for immunohistochemical evaluation of neutrophils present in different regions of the reproductive tract. The numbers indicate the percentage of mice that had positive staining and the severity was a subjective assessment of the percent area of positive staining.

N: not vaccinated; V: EB vaccinated.

## References

[1] CDC (2012). *2011 Sexually Transmitted Disease Surveillance: Chlamydia*.

[2] Brunham R. C., Rey-Ladino J. (2005). Immunology of *Chlamydia* infection: implications for a *Chlamydia trachomatis* vaccine. *Nature Reviews Immunology*.

[3] Hafner L., Beagley K., Timms P. (2008). Chlamydia trachomatis infection: host immune responses and potential vaccines. *Mucosal Immunology*.

[4] Darville T., Andrews C. W., Sikes J. D., Fraley P. L., Rank R. G. (2001). Early local cytokine profiles in strains of mice with different outcomes from chlamydial genital tract infection. *Infection and Immunity*.

[5] Pal S., Peterson E. M., de la Maza L. M. (2005). Vaccination with the chlamydia trachomatis major outer membrane protein can elicit an immune response as protective as that resulting from inoculation with live bacteria. *Infection and Immunity*.

[6] Pal S., Peterson E. M., Rappuoli R., Ratti G., de la Maza L. M. (2006). Immunization with the *Chlamydia trachomatis* major outer membrane protein, using adjuvants developed for human vaccines, can induce partial protection in a mouse model against a genital challenge. *Vaccine*.

[7] O'Connell C. M., Ingalls R. R., Andrews C. W., Scurlock A. M., Darville T. (2007). Plasmid-deficient *Chlamydia muridarum* fail to induce immune pathology and protect against oviduct disease. *The Journal of Immunology*.

[8] Cong Y., Jupelli M., Guentzel M. N., Zhong G., Murthy A. K., Arulanandam B. P. (2007). Intranasal immunization with chlamydial protease-like activity factor and CpG deoxynucleotides enhances protective immunity against genital *Chlamydia muridarum* infection. *Vaccine*.

[9] Farris C. M., Morrison S. G., Morrison R. P. (2010). CD4^+^ T cells and antibody are required for optimal major outer membrane protein vaccine-induced immunity to *Chlamydia muridarum* genital infection. *Infection and Immunity*.

[10] Miyairi I., Ramsey K. H., Patton D. L. (2010). Duration of untreated *Chlamydial genital* infection and factors associated with clearance: review of animal studies. *Journal of Infectious Diseases*.

[11] de la Maza L. M., Pal S., Khamesipour A., Peterson E. M. (1994). Intravaginal inoculation of mice with the *Chlamydia trachomatis* mouse pneumonitis biovar results in infertility. *Infection and Immunity*.

[12] Morrison R. P., Caldwell H. D. (2002). Immunity to murine chlamydial genital infection. *Infection and Immunity*.

[13] Shah A. A., Schripsema J. H., Imtiaz M. T. (2005). Histopathologic changes related to fibrotic oviduct occlusion after genital tract infection of mice with *Chlamydia muridarum*. *Sexually Transmitted Diseases*.

[14] Stephens R. S. (2003). The cellular paradigm of chlamydial pathogenesis. *Trends in Microbiology*.

[15] Darville T., Hiltke T. J. (2010). Pathogenesis of genital tract disease due to *Chlamydia trachomatis*. *Journal of Infectious Diseases*.

[16] Lee H. Y., Schripsema J. H., Sigar I. M., Murray C. M., Lacy S. R., Ramsey K. H. (2010). A link between neutrophils and chronic disease manifestations of *Chlamydia muridarum* urogenital infection of mice. *FEMS Immunology and Medical Microbiology*.

[17] Frazer L. C., O'Connell C. M., Andrews C. W., Zurenski M. A., Darville T. (2011). Enhanced neutrophil longevity and recruitment contribute to the severity of oviduct pathology during *Chlamydia muridarum* infection. *Infection and Immunity*.

[18] Wiesenfeld H. C., Heine R. P., Krohn M. A. (2002). Association between elevated neutrophil defensin levels and endometritis. *Journal of Infectious Diseases*.

[19] Riley M. M., Zurenski M. A., Frazer L. C. (2012). The recall response induced by genital challenge with *Chlamydia muridarum* protects the oviduct from pathology but not from reinfection. *Infection and Immunity*.

[20] Kossodo S., Zhang J., Groves K. (2011). Noninvasive *in vivo* quantification of neutrophil elastase activity in acute experimental mouse lung injury. *International Journal of Molecular Imaging*.

[21] Ho A.-S., Chen C.-H., Cheng C.-C. (2014). Neutrophil elastase as a diagnostic marker and therapeutic target in colorectal cancers. *Oncotarget*.

[22] Jaffer F. A., Libby P., Weissleder R. (2009). Optical and multimodality molecular imaging: insights into atherosclerosis. *Arteriosclerosis, Thrombosis, and Vascular Biology*.

[23] Nahrendorf M., Sosnovik D. E., French B. A. (2009). Multimodality cardiovascular molecular imaging, part II. *Circulation: Cardiovascular Imaging*.

[24] Chen J., Tung C.-H., Mahmood U. (2002). In vivo imaging of proteolytic activity in atherosclerosis. *Circulation*.

[25] National Research Council (U.S.) (2011). *Guide for the Care and Use of Laboratory Animals*.

[26] Wooters M. A., Kaufhold R. M., Field J. A., Indrawati L., Heinrichs J. H., Smith J. G. (2009). A real-time quantitative polymerase chain reaction assay for the detection of *Chlamydia* in the mouse genital tract model. *Diagnostic Microbiology and Infectious Disease*.

[27] American Veterinary Medical Association (2013). *AVMA Guidelines for the Euthanasia of Animals: 2013 Edition*.

[28] Zhang H., Zhou Z., Chen J. (2014). Lack of long-lasting hydrosalpinx in A/J mice correlates with rapid but transient chlamydial ascension and neutrophil recruitment in the oviduct following intravaginal inoculation with *Chlamydia muridarum*. *Infection and Immunity*.

[29] Gupta R., Wali S., Yu J.-J. (2014). *In vivo* whole animal body imaging reveals colonization of *Chlamydia muridarum* to the lower genital tract at early stages of infection. *Molecular Imaging and Biology*.

[30] Campbell J., Huang Y., Liu Y., Schenken R., Arulanandam B., Zhong G. (2014). Bioluminescence imaging of *Chlamydia muridarum* ascending infection in mice. *PLoS ONE*.

[31] Darville T., Andrews C. W., Laffoon K. K., Shymasani W., Kishen L. R., Rank R. G. (1997). Mouse strain-dependent variation in the course and outcome of chlamydial genital tract infection is associated with differences in host response. *Infection and Immunity*.

[32] Chen J., Yang Z., Sun X. (2015). Intrauterine infection with plasmid-free *Chlamydia muridarum* reveals a critical role of the plasmid in chlamydial ascension and establishes a model for evaluating plasmid-independent pathogenicity. *Infection and Immunity*.

[33] Woolridge R. L., Grayston J. T., Chang I. H., Cheng K. H., Yang C. Y., Neave C. (1967). Field trial of a monovalent and of a bivalent mineral oil adjuvant trachoma vaccine in Taiwan school children. *American Journal of Ophthalmology*.

[34] Nichols R. L., Bell S. D., Murray E. S., Haddad N. A., Bobb A. A. (1966). Studies on trachoma. V. Clinical observations in a field trial of bivalent trachoma vaccine at three dosage levels in Saudi Arabia. *American Journal of Tropical Medicine and Hygiene*.

[35] Sowa S., Sowa J., Collier L. H., Blyth W. A. (1969). Trachoma vaccine field trials in the Gambia. *Journal of Hygiene*.

