# Supplementary material for: Quantitative In Vivo Detection of Chlamydia muridarum Associated Inflammation in a Mouse Model Using Optical Imaging
Source: Mediators Inflamm. 2015 Nov 18;2015:264897. doi: 10.1155/2015/264897 (PMC4667028; doi:10.1155/2015/264897)
Supplement: Supplementary file 1 — A detailed histopathologic summary of findings in reproductive tracts of female mice infected with C. muridarum. Mice were sacrificed at the indicated time point and the reproductive tracts were prepared for histopathology assessment as described in the methods. The data presented here is a compilation of several studies. The numbers indicate the percentage of mice that had findings. Severity was graded semi-quantitatively on a scale of 1 to 5 according to routine procedures (1=very slight, 2=slight, 3=moderate, 4=marked, and 5=severe). Acute inflammation was mostly neutrophilic while chronic inflammation was lymphocytic in nature. [file 264897.f1.pdf]

Table S1

Incidence (%) and Severity of Histomorphologic Findings in Mice Infected with *C. muridarum*								
Days post-infection	7		10		14		21	
Vaccination Status	N	V	N	V	N	V	N	V
# Mice evaluated	15	5	5	5	25	9	15	5
**Ovarian Bursa**								
Inflammation-acute	67	40	80	60	96	-	7	-
	vs-m	m	m-mk	m	s-mk		s	
Inflammation-chronic	-	-	-	-	-	11	67	40
						s	vs-s	s
Dilatation	13	60	100	60	72	-	-	20
	vs-s	vs-s	vs-s	vs-s	vs-m			vs
**Oviduct**								
Inflammation-acute	60	40	80	60	96	-	7	-
	vs-m	m	mk	m-mk	vs-mk		vs	
Inflammation-chronic	-	-	-	-	-	-	87	40
							vs-m	vs-s
Dilatation	7	20	80	60	32	22	53	-
	vs	vs	s-m	vs-s	vs-s	vs-m	vs-m	
**Uterus**								
Inflammation-acute	87	60	100	80	92	-	-	-
	vs-s	vs-s	vs-m	vs-s	vs-m			
Inflammation-chronic	-	-	-	-	-	11	100	40
						vs	vs-m	vs-s
Cystic Endometrium	-	-	20	-	12	11	47	40
			vs		vs	vs	vs	s
Decidual reaction	-	-	-	-	12	-	40	-
					vs-m		vs-m	
Hemorrhage	-	-	-	-	12	-	27	-
					p		p	

N=not vaccinated; V= EB vaccinated

vs = very slight; s=slight, m=moderate, mk=marked, p=present

- = No noteworthy change

A detailed histopathologic summary of findings in reproductive tracts of female mice infected with *C. muridarum*. Mice were sacrificed at the indicated time point and the reproductive tracts were prepared for histopathology assessment as described in the methods. The data presented here is a compilation of several studies. The numbers indicate the percentage of mice that had findings. Severity was graded semi-quantitatively on a scale of 1 to 5 according to routine procedures (1=very slight, 2=slight, 3=moderate, 4=marked, and 5=severe). Acute inflammation was mostly neutrophilic while chronic inflammation was lymphocytic in nature.
